# Lithium Salt Association-Mediated Interfacial Charge Exchange for Low-Temperature Lithium-Metal Batteries: Beyond Lithium De-Solvation Manner

**DOI:** 10.34133/research.0802

**Published:** 2025-08-04

**Authors:** Fei Zhao, Jin-Hao Zhang, Jin-Xiu Chen, Zhi-Yuan Gu, Xiao-Zhong Fan, Lin Zhu, Hui-Ling Na, Ming-Xia Dong, Cao Guan, Long Kong

**Affiliations:** ^1^ Institute of Flexible Electronics, Northwestern Polytechnical University, Xi’an 710129, China.; ^2^ Air Defense and Antimissile School, Air Force Engineering University, Xi’an 710100, China.; ^3^ CNPC Research Institute of Safety & Environment Technology, Beijing 102206, China.; ^4^ BASF Shanshan Battery Materials Co. Ltd., Changsha 410205, China.

## Abstract

Fairly assessing energy barrier that shifts coordinated lithium (Li) to naked Li on the interface, as well as deeply exploring interfacial descriptors that can interpret rapid interfacial redox kinetics with anion-dominated electrolyte species, has been long-standing fundamentals to design well-performing electrolytes for low-temperature Li metal batteries. The Li de-solvation concept is merely a picture that can describe the transformation of coordinated Li to naked Li. This work highlights the importance of Li de-coordination instead of Li de-solvation to illustrate such Li transformation behavior, since it considers entire Li de-sheath events (both solvent and anion). Theoretical calculations inform that anions entering into the first Li solvation sheath (mimic to the weak solvation electrolyte) unavoidably elevate the Li de-coordination energy due to the intrinsically greater ion–ion than ion–dipole interactions in the bulk electrolyte. The subsequent interfacial model suggests that interfacial charge exchange is a more effective descriptor to mediate interfacial redox kinetics and interpret experimental results that anion-rich Li species exhibit better battery performances. This work underscores anion effects on the Li de-coordination in the bulk electrolyte and charge exchange in the interface, hoping to unveil the fundamental causes why anion-prevailed Li species work well in low-temperature Li metal batteries.

## Introduction

Lithium (Li) transport across the bulk electrolyte and transfer over electrolyte/electrode interface are placed to be critical in mass and charge migration in working Li-ion batteries [1–4]. In the bulk electrolyte, the solvated Li with a solvent-separated ion pair (SSIP) format is advocated to expedite Li transport under an appropriate electric field, while contact ion pair (CIP) and aggregate (AGG) species bear mild charge density, which hardly migrate between a pair of electrodes [5]. The electrolyte/electrode interface needs to furnish facile transformation from coordinated Li to naked Li, a necessary process to electrochemically plate or insert Li on/into electrodes. Li behavior near the interface is scientifically more interesting than bulky electrolyte in the domain of battery chemistry, as the transformation of coordinated to naked Li is generally deemed as the rate-limiting step, especially in low-temperature conditions [6,7]. The redox kinetics in Li metal anode is quite severe than insertion mechanism anodes, like silicon [8,9]. For example, the previous work reported a huge increase (more than 2 orders of magnitude) of the charge transfer resistance on the interface compared with room temperature [10]. The importance of charge and mass transfer over electrolyte/electrode interface triggers enormous effects to design suitable electrolyte constituents on the basis of electrolyte solvation chemistry [11–15].

A popular strategy to electrochemically propel Li plating from electrolyte to interface under low temperature is to screen solvents with week solvation ability, such that 2-fold attributes are plausible explanations to rationalize the enhanced battery performances, considering ion–solvent interactions and protective layer properties [16–19]. (a) The anion is introduced into the first sheath of Li^+^, which benefits the solid electrolyte interphase (SEI) with inorganic-rich components (e.g. LiF, Li_2_O, and so on) [20–23]. These anion-derived inorganic species are particular interests in recent studies, since they are supposed to facilitate Li transport across the SEI [24–27]. (b) The interaction between Li^+^ and solvents is largely tamed, resulting in the so-called low “de-solvation” energy, contributing to the reduced electrochemical overpotential [28]. The concept of de-solvation is so frequently used in the weak solvation electrolyte that the anion binding strength in the coordinated Li environment is often overlooked in the interpretation of enhanced low-temperature battery performances. However, once strong donicity anions, such as nitrate (NO_3_^−^) and difluoro(oxalato)borate (DFOB^−^), move into the first sheath of Li^+^ and part of coordinated solvents are squeezed out, the “de-solvation energy” with reduced number of coordinated solvents around Li^+^ is merely a picture that can exactly depict low barrier of shifting coordinated Li to naked Li, since it ignores the event that the anion also tightly binds with Li cations [29–31].

The term “hydration” is the predecessor of “solvation” in physical chemistry, the latter of which is thereafter the descriptor of interactions that takes place when an ion is introduced into solvents (including both water and inorganic solvents) [32]. Gurney defines the solvation as a term that is associated with the interaction between a solute and a solvent. Therefore, the “de-solvation” is a microscopic process that must include the solvent behavior, whereas de-binding anions from the cation center is rarely considered in the CIP and AGG dominated weak solvation electrolyte system, resulting in rather underestimated energy that transforms the coordinated Li to naked Li [33]. To this line, Li coordination is a broader concept than Li solvation, since the former not only includes the interaction between Li and solvents but also considers the interaction between Li and anions. This refined description underlines the term “de-coordination” instead of “de-solvation” to more accurately interpret the Li transformation from coordinated to naked Li states.

To date, there has been an understanding gap between experimental results and theoretical insights: Experimentally, the electrolyte with a relatively strong coordinated anion (mimic to the weak solvation electrolyte with anions entering the first Li solvation sheath) generally exhibits better kinetic features in Li batteries under low temperature, while theoretically, such anions impair electrolyte ionic conductivity and elevate the Li de-coordination barrier, both of which principally retard the redox kinetics of battery operations [34,35].

In this work, the term “de-coordination” is highlighted to interpret Li plating behavior from electrolyte to electrode when the Li is coordinated with strong binding anions in the first solvation sheath. Moreover, the interfacial charge exchange is suggested to be a fresh viewpoint to rationalize the conclusion that high overall Li coordination environment does not necessarily imply greater overpotential to shift coordinated to naked Li on the interface. The interfacial charge exchange describes the process that the anion-involved Li solvates chemically adsorb the interface and thus trigger the electron exchange between coordinated Li and anode surface. The high-donicity anion contributes to the Li–anion association that theoretically facilitates the interfacial charge exchange during the surface redox reactions, which is the critical cause of experimentally enhanced exchange current density and low charge transfer resistance at low temperature. As the case of verification in the anion strength-mediated redox kinetics, Li||NCM622 cells with a high mass loading (28.27 mg cm^−2^, corresponding to an areal capacity of 4.5 mAh cm^−2^) deliver an impressive cycling stability electrolyte (retaining 1.7 mAh cm^−2^ after 200 cycles at low temperature) and rate capacity (1 mAh cm^−2^ at 0.8 C). This work bridges the understanding gap between high Li de-coordination energy and expedited Li plating kinetics under low temperature, and hopes to inspire innovative design principles of electrolyte constituents for energy-dense and temperature-robust Li metal batteries.

## Results and Discussion

### Li salt association and transport behavior

To investigate the Li de-coordination energy influenced by anion donicity, lithium hexafluorophosphate (LiPF_6_) and lithium difluoro(oxalate)borate (LiDFOB) with diverse coordination strengths are selected as model Li salts that dissolved in fluoroethylene carbonate (FEC) and methyl ethyl carbonate (EMC) with v/v = 1/4. Anion binding strength to Li^+^ is considered to impact the entire Li de-coordination strength. An intuitive sense of high anion binding strength leads to the high Li de-coordination energy to impede the Li plating kinetics, whereas the final results seem to be rather comprehensive.

**Fig. 1.**
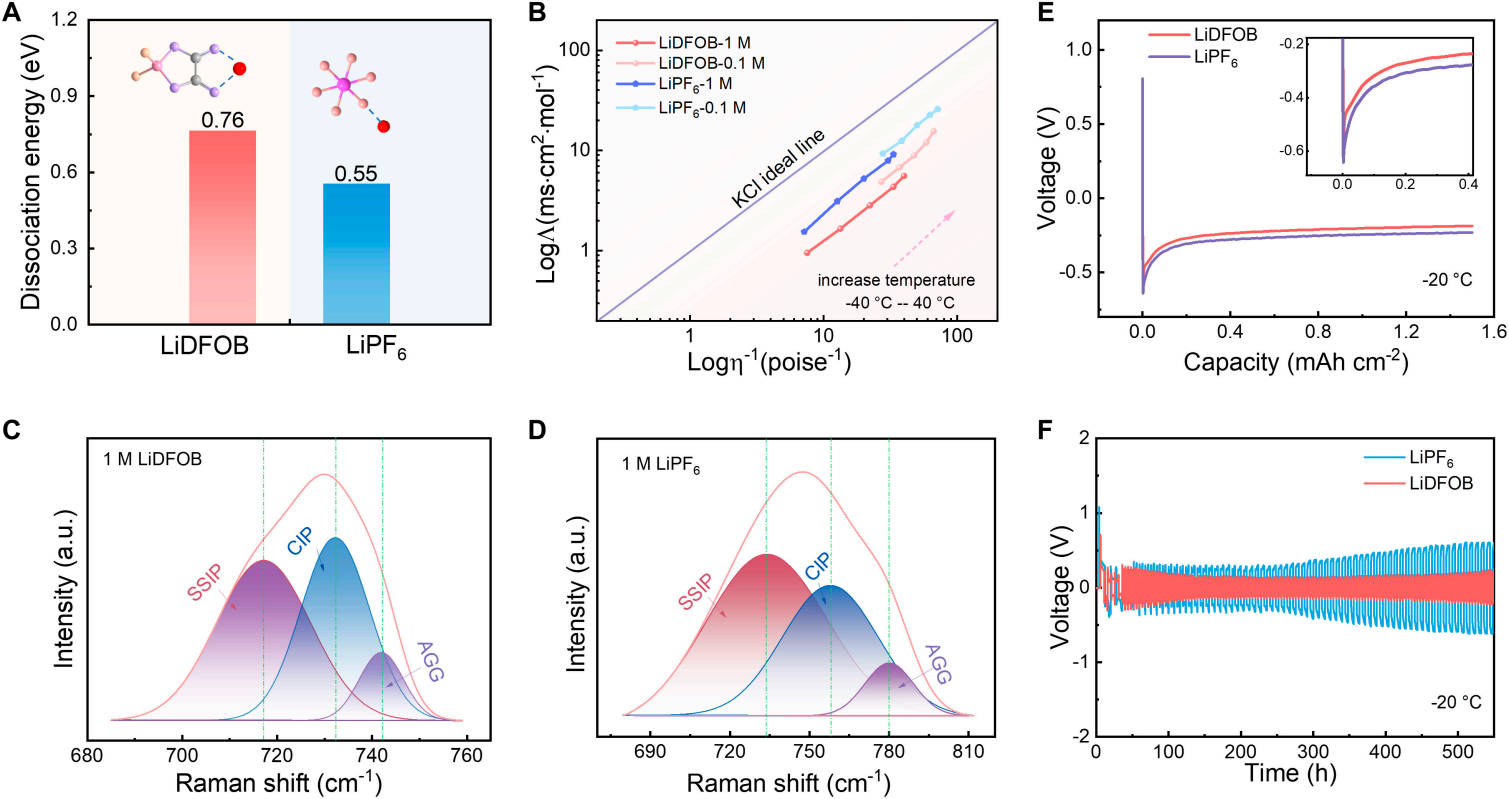
The analysis of Li^+^–solvent coordination. (A) Dissociation energy of LiDFOB and LiPF_6_ in solvents. (B) Walden plot of Li salts under various temperatures. Raman spectra of (C) LiDFOB and (D) LiPF_6_ electrolytes to decouple content of SSIP, CIP, and AGG. (E) Voltage profile of Li plating on the Cu foil in Li||Cu cell. (F) Voltage–time profile of Li||Li symmetric cell at −20 °C.

In order to justify this assumption, LiPF_6_ and LiDFOB are studied, with the coordinating strength of DFOB^−^ being higher than that of PF_6_^−^. The dissociation energies of 2 Li salts under the solvent influence are calculated to be 0.76 and 0.55 eV for LiDFOB and LiPF_6_, respectively (Fig. 1A). Both Li salts require much less energy (approximately one order of magnitude lower) for dissociation in electrolytes compared to the dissociation energy in a vacuum (Fig. S1). Ionic conductivity and electrolyte viscosity at different conditions are shown in Fig. S2. The Li salt dissociation behavior can also be expressed by combining viscosity and molar ionic conductivity (Fig. 1B) [36,37]. The line of ideality in this diagram is determined by 0.01 M KCl solution, which qualitatively evaluates the dissociation degree of Li salts in electrolytes, with approaching the line being more dissociation [38]. As shown in Fig. 1B, the nearly linear correlation with an increase of temperature at different concentrations for the LiPF_6_ electrolytes is close to the standard KCl line, indicating a high dissociation degree. The LiDFOB at the same test conditions is away from the KCl line compared with LiPF_6_, further confirming the high associating energy barrier of LiDFOB. The high LiDFOB associating energy barrier reduces quantity of charge carriers in the electrolyte and impairs the vehicular diffusion under electric field, pushing the Li transportation mode toward structural diffusion [39]. This shift of Li transportation mode contributes to the enhanced transference number at both room and low temperature conditions (Fig. S3).

Raman spectroscopy is a powerful tool to detect electrolyte species of SSIP, CIP, and AGG by deconvoluting Raman line shape [40]. The Raman spectra identify the Li^+^ coordination with solvents and anions in the range of 680 to 810 cm^−1^ in LiPF_6_- and LiDFOB-based electrolytes (Fig. 1C and D). Finely deconvoluting the line shape of 2 electrolytes results in a relatively higher CIP (41.03%) and AGG (9.77%) content in the LiDFOB electrolyte than LiPF_6_ electrolyte (35.59% for CIP and 7.32% for AGG) (Fig. S4). ^7^Li and ^19^F NMR data support this hypothesis and provide additional insights into the coordination structure (Fig. S5). As anticipated, the ^7^Li peak shift toward high field owing to the electron donating by solvents, signifying the impaired interaction between Li^+^ and PF_6_^−^, which indirectly indicates the greater interaction between Li^+^ and DFOB^−^. Such anion coordination strength contributes to a pronounced peak for the Li^+^–DFOB^−^ interaction at 1.67 Å in the radial distribution function (RDF) obtained by molecular dynamics (MD), which significantly dwarfs the Li^+^–PF_6_^−^ profile (Fig. S6). The collective experimental and computational results indicate the strong Li–DFOB interaction in the LiDFOB electrolyte (Fig. S7).

A surprising electrochemical result that can characterize Li plating kinetic features goes against the ramifications of Li–anion association behavior. The initial Li plating overpotential with a more associated LiDFOB salt is lower than that with LiPF_6_ salt in Li||Cu cells at −20 °C (Fig. 1E). The symmetric Li||Li cells also suggest that the LiDFOB-based electrolyte is more compatible with the Li metal anode at both room and low temperature conditions, showing the long-term cyclability and dampened Li plating/striping overpotential (Fig. 1F and Fig. S8). This result challenges the intuitive assumption that high coordination anion requiring extra energy to switch coordinated Li to naked Li would contribute to the greater Li plating overpotential. In the following subsections, this work will discuss why the highdonicity anioninvolved Li solvates benefit Li redox kinetics, with special focus on bulk and interface characteristics.

### Anion-induced Li de-coordination energy in the bulky electrolyte

Assessing and comparing the coordination strength of anions and solvents have been the scientific interests to quantify the electrolyte speciation, such as SSIP, CIP, and AGG. However, there has been no consensus on the parameter that can fairly benchmark their coordination strengths [41]. Dielectric constant has been proposed to indicate the solvent coordination strength, while solvent geometry and denticity in the influence of coordination strength are generally ignored, usually drawing the inaccurate or even incorrect conclusions [42]. In addition, the dielectric constant values regarding diverse anions are inaccessible, resulting in difficulty to compare the coordination strength between the solvents and anions. Although the donor number can be available for both solvents and anions, it does not consider denticity. Especially, the donor number values obtained from antimonic chloride (SbCl_5_) in dilute 1,2-dichloroethane solution cannot directly transfer to Li salts, since the large difference of charge density (0.68 Å for Li^+^ versus 0.62 Å for Sb^5+^) could drastically alter the structure deformation of anions [43].

Electrostatic potential (ESP) is a common measure of charged groups [44]. ESP is induced on the surface of a molecule by its nuclei and electrons, and reflects an unbalanced distribution of charge [45]. The most positive potential (ESP_max_) and the most negative potential (ESP_min_) could be used to measure the polarity of a molecule. Since the Li–anion and Li–solvent interactions are dominantly influenced by the electrostatic interaction, ESP_min_ is well suited to qualitatively indicate the interaction strength of solvents and anions toward Li cation in electrolytes, with lower ESP_min_ being more tightly binding with Li^+^.

**Fig. 2.**
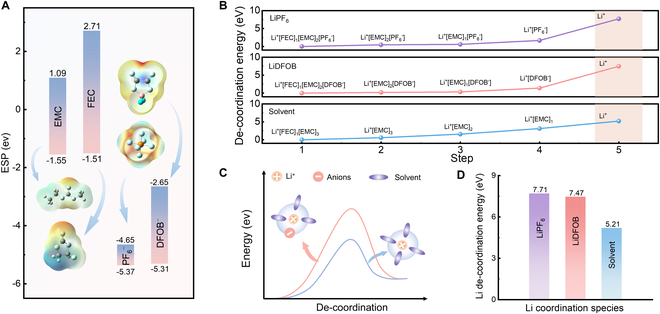
The coordination behavior of Li^+^. (A) ESP and surface field of solvents and anions. (B) Step-by-step de-coordination energy of entire Li de-sheath events (both solvent and anion). (C) Schematic diagram of Li de-coordination energy with and without anion in the first coordination sheaths. (D) Quantitative comparison of Li de-coordination energies with different anions and solvents presented in the first coordination sheaths.

The ESP values of FEC, EMC, LiPF_6_, and LiDFOB are calculated, as shown in Fig. 2A. The solvent polarity can be clearly identified, with the negative charge surrounding carbonyl oxygen [46,47]. These negative charged moieties bind with positively charged Li^+^, while the positively charged moieties of solvents may weakly interact with other solvents through dipole–dipole interactions. It is worth noting that even the ESP_max_ of both anions is still lower than the ESP_min_ of FEC and EMC solvents, unambiguously suggesting the strong electrostatic interaction between Li and anions. It is also the root cause that very diluted electrolytes with Li concentration less than 0.01 M also contain CIP [48,49]. Once the anion enters the first coordination sheath, the strong electrostatic interaction contributes to a greater de-coordination profile (Fig. 2B and C) according to Li coordination environment (Fig. S9 and Table S1). This prediction is further verified by calculating the de-coordination (Δ*E*_de-coord_) energy using solvation model that considers different numbers of solvents and anions in the first solvation sheath (namely, Li[FEC][EMC]_2_[PF_6_], Li[FEC][EMC]_2_[DFOB], and Li[FEC][EMC]_3_), as shown in Fig. 2D. The results reveal that the Δ*E*_de-coord_ of Li^+^ with respect to PF₆^−^ and DFOB^−^ are 7.71 and 7.47 eV, much higher than that of Li^+^ with entire solvents (5.21 eV). The ESP of solvents and anions, as well as calculated Li de-coordination energy, indicates the importance of anion binding when shifting the coordinated to naked Li^+^.

The above analysis of Li solvation behavior in the bulk electrolyte could draw the following 2 critical conclusions. First, negatively charged anions that enter the first solvation sheath could profoundly elevate the Li de-coordination energy. The conventionally used de-solvation energy (normally claimed low de-solvation energy) underestimates the required energy that shifts coordinated Li^+^ to naked Li^+^. Second, the stronger anion binding strength does not necessarily imply the higher Li de-coordination energy. For example, the dissociation energies of LiDFOB and LiPF_6_ are 0.76 and 0.55 eV, respectively, while the Li de-coordination energies are 7.47 and 7.71 eV, correspondingly. The underlying causes to rationalize this difference are still elusive, requiring further computational and experimental studies. The possible explanations are the steric effects of bulky anion, as well as entropy contributions [50].

### Benefiting interfacial charge exchange by Li salt association

Li de-solvation behavior on the interface is another key influencer to dictate the Li plating kinetics during interfacial redox reactions. To interpret the high Li salt association that has been experimentally corroborated to benefit the dampened Li plating/striping overpotential, the interface charge exchange is considered, since the charge accumulation around the interface leads to an increase of the ion density near the interface, causing the charge redistribution of the solvent and ions, which in turn influences the ion diffusion behavior, Li de-coordination process, and the kinetics of electrochemical reactions at the interface [51,52].

**Fig. 3.**
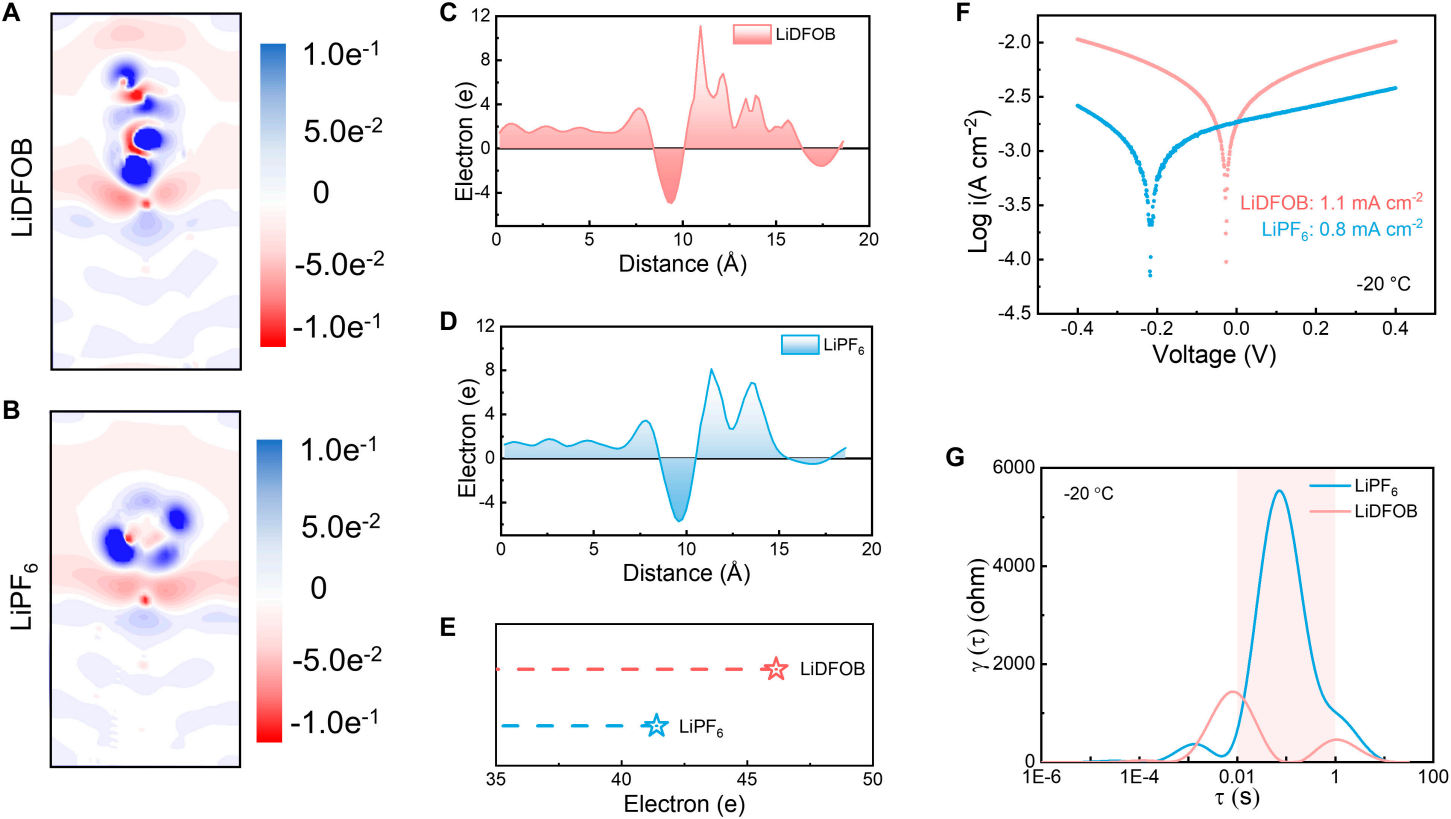
Anion influenced surface charge distribution and kinetics. 2D slices of charge density difference of (A) LiDFOB and (B) LiPF_6_ solvated structures on Li surface. Space charge density difference function influenced by (C) LiDFOB and (D) LiPF_6_. (E) Quantitative analysis of effective charges in the near-surface region. (F) Exchange current density derived from Tafel test at low temperature. (G) Decoupling kinetic processes by DRT to identify resistive behaviors at low temperature.

The Li salt association aids the anion-dominated coordination and thus alter the charge density on the Li metal surface. The large interface charge exchange implies the more intensive interaction between electrolyte species and anode surface. This demanding interface charge exchange is the prerequisite step to initiate follow-up charge and mass exchange during redox [53]. The charge density difference and the corresponding 2-dimensional (2D) slices are computed via density functional theory (DFT) to elaborate the charge redistribution. Red region indicates the accumulation of negative charges on the surface of the Li metal anode, and the blue region represents the areas where positive charges are enriched (Fig. 3A and B). By integrating the spatial distribution of positive and negative charges over the area that covers a 20-Å range on the surface of the Li metal anode, the Li anode surface with the LiDFOB electrolyte exhibits a remarkable charge accumulation compared with the one with LiPF_6_ (Fig. 3C and D). This spatial charge distribution originates from the Li^+^–DFOB^−^ association, which prompts more anions to directionally aggregate at the electrode/electrolyte interface. Quantitative analysis reveals that the LiDFOB-based electrolyte accumulates 46 effective charges in the near-surface region, while the surface region with LiPF_6_ electrolyte has only 42 effective charges (Fig. 3E). This Li salt association dependence of charge density is considered as the vital factor that leads to the different Li plating/stripping overpotential.

The Li salt association-mediated interface charge exchange is expected to hasten redox kinetics of interface reactions. The Tafel curve provides a more direct comparison of Li plating kinetics. Fitting the Tafel curve (Fig. 3F) informs a larger exchange current density (1.1 mA cm^−2^) of the LiDFOB electrolyte than LiPF_6_ electrolyte (0.8 mA cm^−2^) at −20 °C, indicating an accelerated kinetics for Li deposition [54]. Notably, the LiDFOB-based electrolyte maintained a distinct advantage at room temperature (Fig. S10). The resistive behavior as the function of time scale could unambiguously decouple ohmic, charge transfer, and mass transport resistance by the distribution of relaxation times (DRT) technique in Li||Li symmetric cells [55]. As the temperature decreases, the dominant factor of the surface resistance shifts from SEI to charge transfer resistance (Fig. 3G and Fig. S11). The activation energy of Li^+^ charge transfer with LiDFOB electrolyte on the Li anode interface is also lower than LiPF_6_ (Fig. S12 and Table S2). The intensive interface exchange current has been proved to facilitate Li^+^ charge transfer.

Based on the above analysis, it can be concluded that the enhanced redox kinetics on the interface is achievable despite the high Li salt association with strong anion binding strength. The expedited Li redox kinetics is intimately correlated to the interface charge exchange that can be elevated by Li association around the interface.

### Battery performances directed by Li salt association

**Fig. 4.**
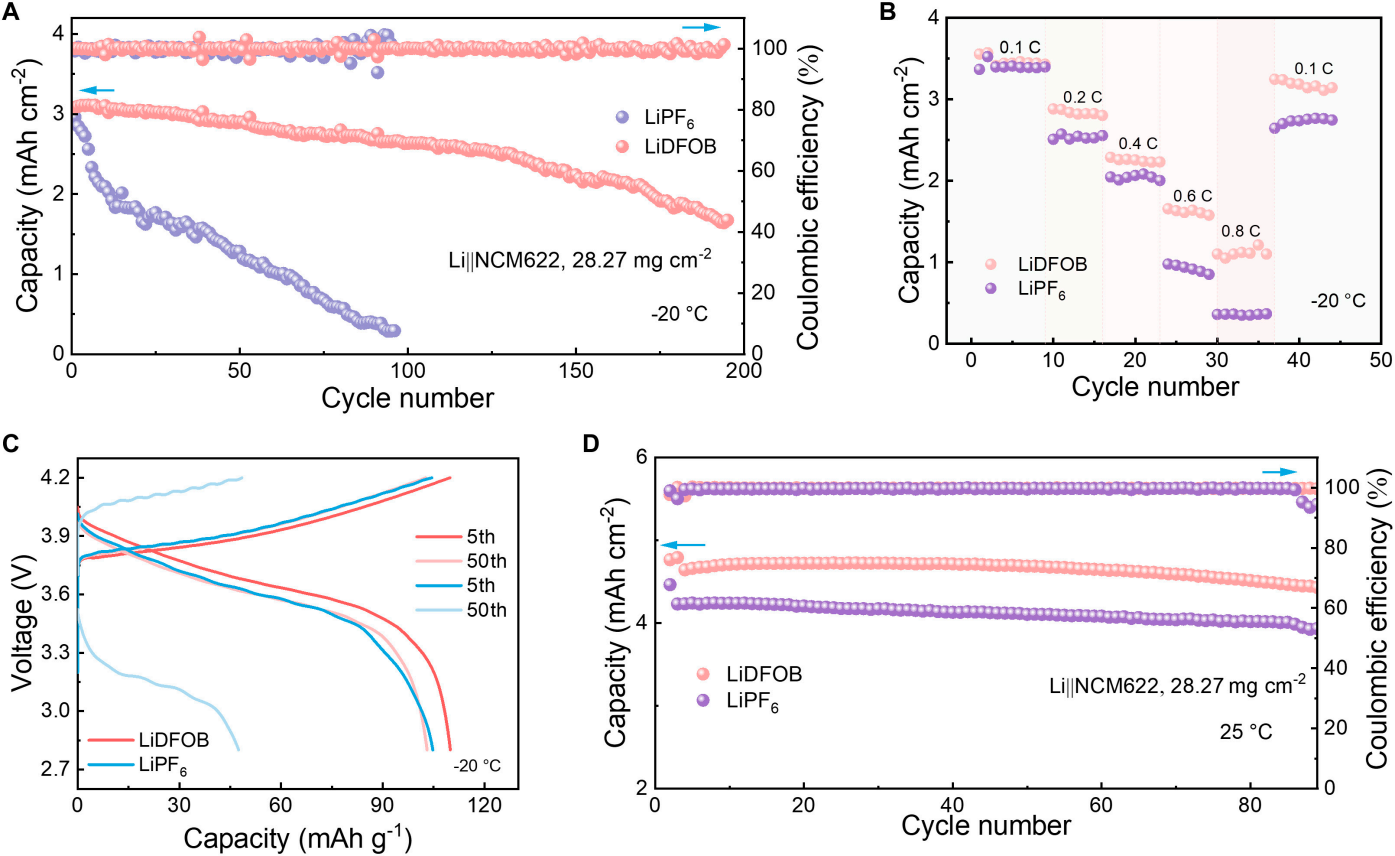
Electrochemical performance of battery electrolytes over a wide temperature range. (A) Cyclic stability of Li||NCM622 cells between 2.8 and 4.2 V with 0.1 C charge and 0.2 C discharge. (B) Rate capability and (C) typical charge/discharge curves of Li||NCM622 cells at low temperature. (D) Cycling stability of Li||NCM622 cells at 25 °C.

In order to evaluate the effect of Li salt association on battery performance, the Li||NCM622 cells with the proposed electrolytes were tested at −20 °C (Fig. 4A) at a high areal capacity of 4.5 mAh cm^−2^, corresponding to an areal mass loading of 27 mg cm^−2^. The cell with the LiDFOB-based electrolyte can be operated for more than 200 cycles at a current density of 1.8 mA cm^−2^. However, the cell with LiPF_6_-based electrolyte exhibits a sharp capacity decay attributed to the sluggish redox kinetics at the interface, causing tremendous irreversible capacity loss, as well as possible Li dendritic growth due to large overpotential [56–58]. In addition, remarkable rate capability is achieved under low temperature with the aid of the LiDFOB-based electrolyte (Fig. 4B), delivering an area capacity of 1 mAh cm^−2^ at 0.8 C, while the cell with the LiPF_6_-based electrolyte cannot work at 0.8 C.

The cell with LiDFOB-based electrolyte demonstrates excellent rate performance at room temperature, maintaining an area capacity of 3.1 mAh cm^−2^ at 1.2 C (Fig. S13). Upon examining charge and discharge curves over different cycles, the LiPF_6_-based cell exhibits appreciable capacity decay after 50 cycles compared with the LiDFOB-based cell (Fig. 4C and Fig. S14). Meanwhile, the LiDFOB-based cell can still maintain a high capacity and high Coulombic efficiency after 90 cycles at room temperature, while the Coulombic efficiency of the cell with LiPF_6_-based electrolyte falls below 90% (Fig. 4D and Fig. S15).

The electrochemical performance of the cells equipped with high coordination-strength anion are markedly improved compared with the LiPF_6_-based electrolyte, unambiguously stressing the importance of Li de-coordination on the interface mediated by ion association. The electrolyte with strong ion–ion interactions promotes the anion accumulation on the interface, which enhances the interface charge exchange, and effectively accelerates ion transport kinetics at low temperatures.

## Conclusion

Li de-coordination is a broader concept than de-solvation to describe transformation from coordinated Li to naked Li, as it considers both omitting solvents and anions around the first Li^+^ sheath. Once the anion enters into the first Li sheath, the Li de-coordination energy is higher than the case that Li is entirely coordinated by neat solvents, as the Li–anion interaction is intrinsically higher than Li–solvents, showing the Li de-coordination energies of 5.21 and 7.47 eV for Li[FEC][EMC]_3_ and Li[FEC][EMC]_2_[DFOB], respectively. In addition, Li de-coordination energy is not strictly correlated with the anion coordination strength, probably due to the contributions from steric effect and entropy variation. For example, the greater tendency of Li association salt (LiDFOB) exhibits slightly lower Li de-coordination energy than that of easily disassociated Li salt (LiPF_6_). To further rationalize the rapid redox kinetics in the case that anion-rich Li solvates dominate electrolyte species in the surface, interface charge exchange is proposed to measure interactions between Li species and anode surface. It reveals an intensive interface charge exchange with enriching anion quantity, which expedites Li redox kinetics in the low temperature conditions. The cell with a high areal loading (27 mg cm^−2^) of NCM622 exhibits considerable improvement in cyclability and rate capability. This work highlights the importance of Li de-coordination concept to describe the Li transformation process from coordinated to naked Li^+^, as well as reveals the importance of interface charge exchange to depict how anion propels Li migration across the interface.

## Materials and Methods

### Chemicals

Ultra-thin and thick Li metal foils (Ke lude), LiPF_6_ (Dodo Chem), LiDFOB (Dodo Chem), FEC (Dodo Chem), ethyl methyl carbonate (EMC; Adamas), polyvinylidene difluoride (PVDF; Dodo Chem), *N*-methyl-2pyrrolidone (NMP; Adamas), LiNi_0.6_Co_0.2_Mn_0.2_O_2_ (NCM622; Ke lude), and *N*,*N*-dimethylformamide (DMF; Adamas) were obtained as received.

### Preparation of LiNi_0.6_Co_0.2_Mn_0.2_O_2_ cathode

The cathode was prepared by mixing NCM622, conductive carbon, and PVDF binder with a mass ratio of 95:3:2 in NMP. The mixtures were milled by a mortar for 60 min. Slurries of cathode were coated on Al foils and then dried for 12 h under vacuum at 80 °C. The normal areal mass loadings of the cathode were approximately 27 mg cm^−2^ (corresponding to 4.5 mAh cm^−2^).

### Preparation of electrolytes

All reagents and Li salts are used as received without purification. LiPF_6_ and LiDFOB (1.0 M) were dissolved in the mixture of FEC/EMC with a volume ratio of 1:4. All these electrolytes are obtained under an argon-filled glove box [O_2_ < 0.1 parts per million (ppm) and H_2_O < 0.1 ppm]. All the electrochemical performance shown in Fig. 4 are acquired in Li||NCM622 cells with 70 μl of electrolyte per cell. The area capacity of NCM622 is about 4.5 m Ah cm^−2^. The ratio of electrolyte to capacity (E/C) is 14 μl mAh^−1^.

### Electrochemical measurements

NEWARE electrochemical testing systems are used to obtain the cycle and rate performances. CHI analytical electrochemical workstation is used to analyze electrochemical performances. NCM622||Li cells are performed with a cutoff voltage of 4.2 V (versus Li/Li^+^). All the cells are fabricated in the argon-filled glovebox with H_2_O and O_2_ < 0.1 ppm. The ionic conductivity measurement is conducted by FE38-Standard meter (METTLER TOLEDO) from 40 to −20 °C in Thermostat (METTLER TOLEDO). The viscosity measurement is conducted by NDJ-5S-8S-9S Digital Viscometer. Electrochemical impedance spectroscopy (EIS) and Tafel plots were all conducted in Chi670e. In particular, the temperature-dependent EIS was performed with a 5-mV voltage amplitude in the frequency range of 10^5^ to 10^−2^ Hz in Li||Li symmetric cells from 253 to 298 K. The activation energy derived from the temperature-dependent Nyquist plots was based on the Arrhenius equation. The Tafel curves were obtained by plotting the overpotential of galvanostatic Li stripping/plating versus the natural logarithm of the current density.

The Li^+^ transference number was determined by the Bruce–Vincent method. Specifically, a small constant voltage (Δ*V*) of 5 mV was applied to the Li||Li symmetric cell to detect the initial current (*I*_ini_) and the steady-state (*I*_ss_) current. The initial (*R*_ini_) and steady-state (*R*_ss_) interfacial resistances were measured by EIS over a frequency range from 10^5^ to 10^−2^ Hz. Finally, the Li^+^ transference number can then be calculated by Eq. 1:

$$t_{+}=\frac{I_{\mathrm{ss}}*\left(∆V-I_{\mathrm{ini}}*R_{\mathrm{ini}}\right)}{I_{\mathrm{ini}}*\left(∆V-I_{\mathrm{ss}}*R_{\mathrm{ss}}\right)}$$ (1)

The activation energy of the Li^+^ de-coordination process was obtained from the temperature-dependent EIS Nyquist plots from 253 to 313 K, fitted by the Arrhenius equation:

$$\ln\left(\frac{T}{R_{\mathrm{ct}}}\right)=\ln A-\frac{E_{a}}{\mathrm{RT}}$$ (2)

where *R*_ct_ is the Li^+^ de-coordination resistance at the interphase, *T* is the absolute temperature, *R* is the gas constant, and *A* is the preexponential factor.

### Characterizations

^7^Li and ^19^F spectra are collected by nuclear magnetic resonance (NMR) spectroscopy on a 600-MHz DirectDrive2 spectrometer. Raman spectroscopy was conducted on the Thermo Fisher DXR Raman spectrometer.

### DFT calculations

DFT calculations were performed in Gaussian 09W to conduct analysis of the structure. B3LYP method and 6-311+G (d, p) basis set were chosen. The solvation effect was considered with the universal solvation model of SMD [59]. The self-consistent field (SCF) convergence on energy is 10^−6^ Hartree. Frequency analysis was performed to ensure the ground state of optimized molecules.

The binding energy was calculated using the same function by Gaussian 09W based on Eq. 3:

$$∆E=E_{\text{product}}-E_{\text{reactant}}$$ (3)

where $E_{\text{product}}$ and $E_{\text{reactant}}$ represent the energy of products and reactants, respectively.

The density difference was calculated by Castep. Generalized gradient approximation (GGA) of the Perdew–Burke–Ernzerhof (PBE) functional was used as the exchange-correlation functional. The Brillouin zone was sampled with 2 × 2 × 1 K points for surface calculation. All surfaces are based on 3 layers of Li. Then, the anion structures were absorbed on the surface. The cut off energy was set as 456 eV, and structure relaxation was performed until the convergence criteria of energy and force reached 1 × 10^−5^ eV and 0.04 eV Å^−1^, respectively. The vacuum layer of 10 Å was constructed to eliminate interactions between periodic structures of surface models.

### Molecular dynamic simulations

Molecular dynamics (MD) simulations were performed using GROMACS. The time step was fixed to be 1 fs for all simulations. The systems were first equilibrated in an isobaric–isothermal ensemble for 4 ns to maintain a temperature of 298 K and a pressure of 1 atm with time constants of 1 ps. A production run of 6 ns at 298 K in a canonical ensemble under Nose–Hoover thermostat was finally conducted. The simulations were output every 1,000 steps and used for the analysis of the RDF [60].

## Data Availability

The data that support the findings of this study are available from the corresponding authors upon reasonable request.
