# Supplementary material for: Lithium Salt Association-Mediated Interfacial Charge Exchange for Low-Temperature Lithium-Metal Batteries: Beyond Lithium De-Solvation Manner
Source: Research (Wash D C). 2025 Aug 4;8:0802. doi: 10.34133/research.0802 (PMC12320520; doi:10.34133/research.0802)
Supplement: Supplementary 1 — Figs. S1 to S15 Tables S1 and S2 [file research.0802.f1.docx]

Supporting Information

Title

Lithium salt association mediated interfacial charge exchange for low-temperature lithium-metal batteries: Beyond lithium de-solvation manner

**Authors**

Fei Zhao^1†^, Jin-Hao Zhang^1†^, Jin-Xiu Chen^1^, Zhi-Yuan Gu^1^, Xiao-Zhong Fan^1^, Lin Zhu^2^*, Hui-Ling Na^3^, Ming-Xia Dong^4^, Cao Guan^1^*, Long Kong^1^*

**Affiliations**

^1^Institute of Flexible Electronics, Northwestern Polytechnical University, Xi'an, 710129, China.

^2^Air Defense and Antimissile School, Air Force Engineering University, Xi'an, 710100, China.

^3^CNPC Research Institute of Safety & Environment Technology, Beijing, 102206, China.

^4^ BASF Shanshan Battery Materials Co., Ltd.

^*^Address correspondence to: amlkong@nwpu.edu.cn (L.K.); iamcguan@nwpu.edu.cn (C.G.); zhulin_chem@126.com (L.Z.)

†These authors contributed equally to this work.


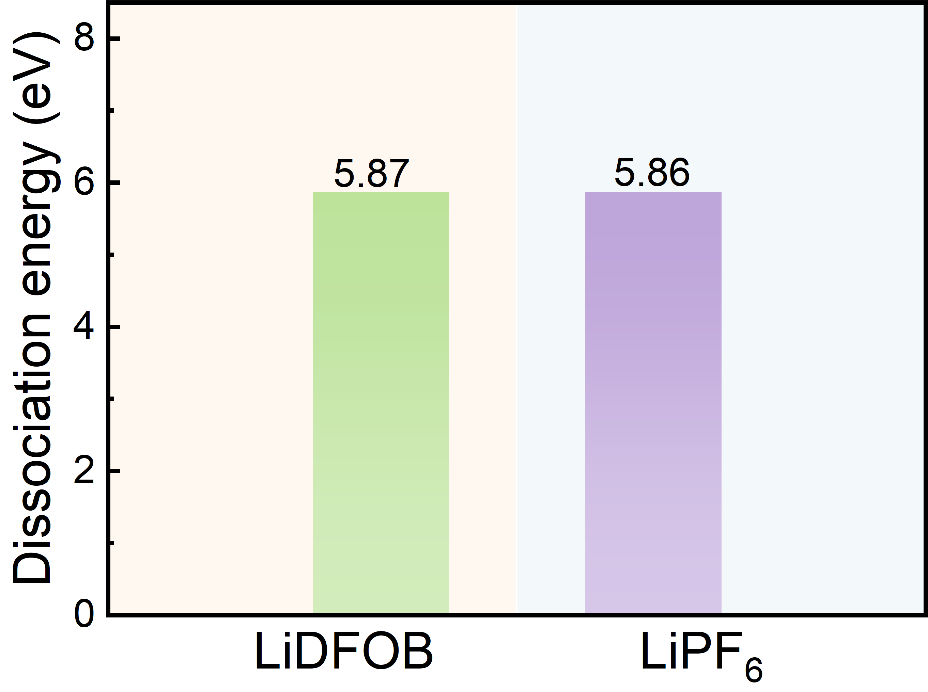


Figure S1. Dissociation energy of lithium salts in vacuum.


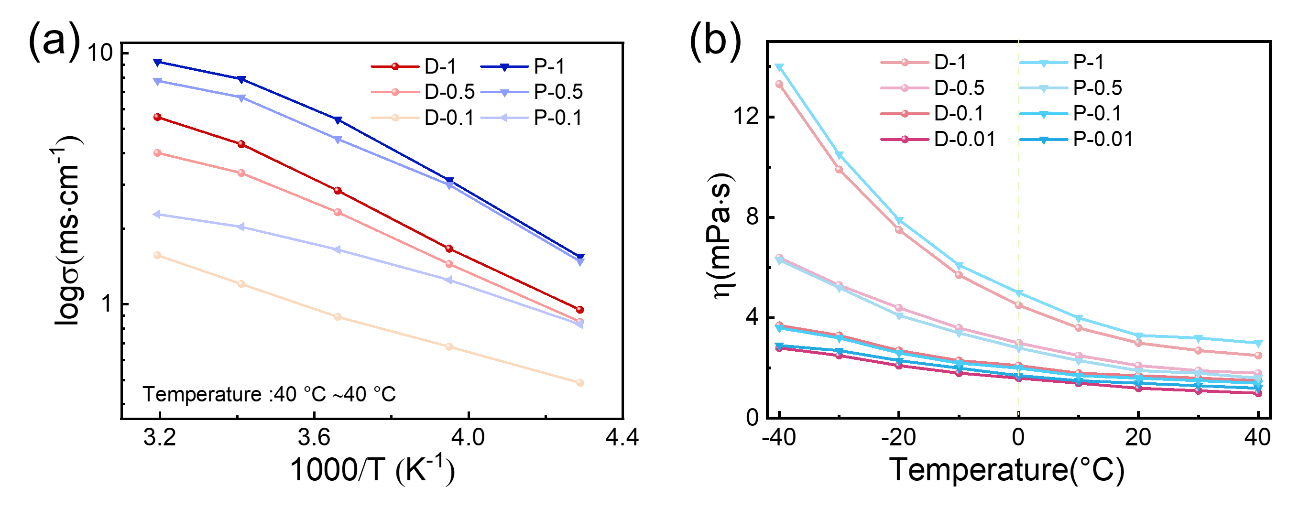


Figure S2. Ionic conductivity and electrolyte viscosity at different concentrations from −40 ºC to 40 ºC. (a) Logarithmic conductivity and (b) viscosity of electrolytes against temperature. D and P represents LiDFOB and LiPF6, respectively. The values indicate the Li salt concentration. For example, D-0.5 means 0.5 M LiDFOB.


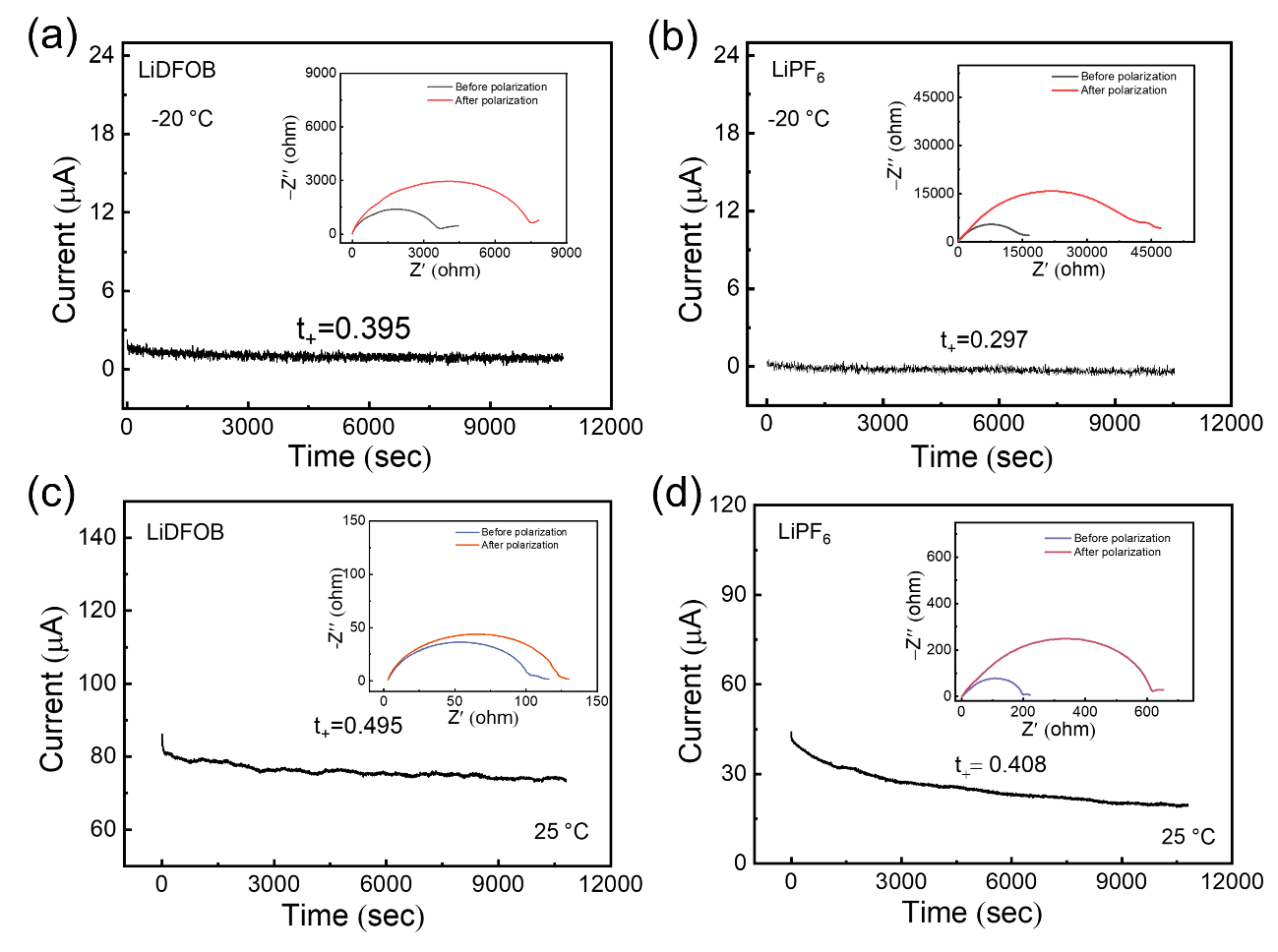


Figure S3. Li^+^ transference number in LiDFOB and LiPF_6_ based electrolytes with (a, b) room temperature and −20 ºC (c, d).


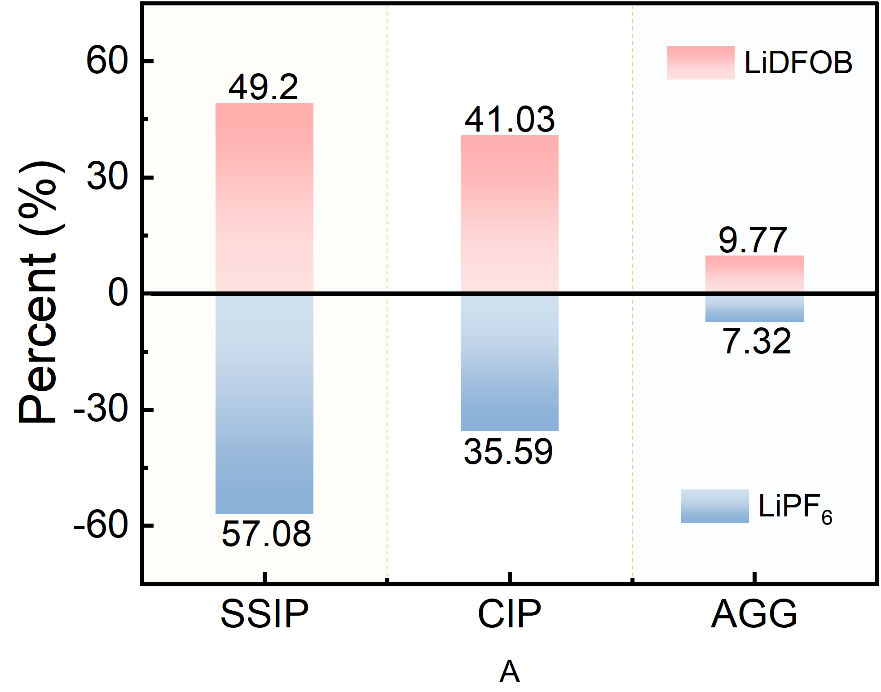


Figure S4. Proportion of electrolyte species derived from Raman spectroscopy.


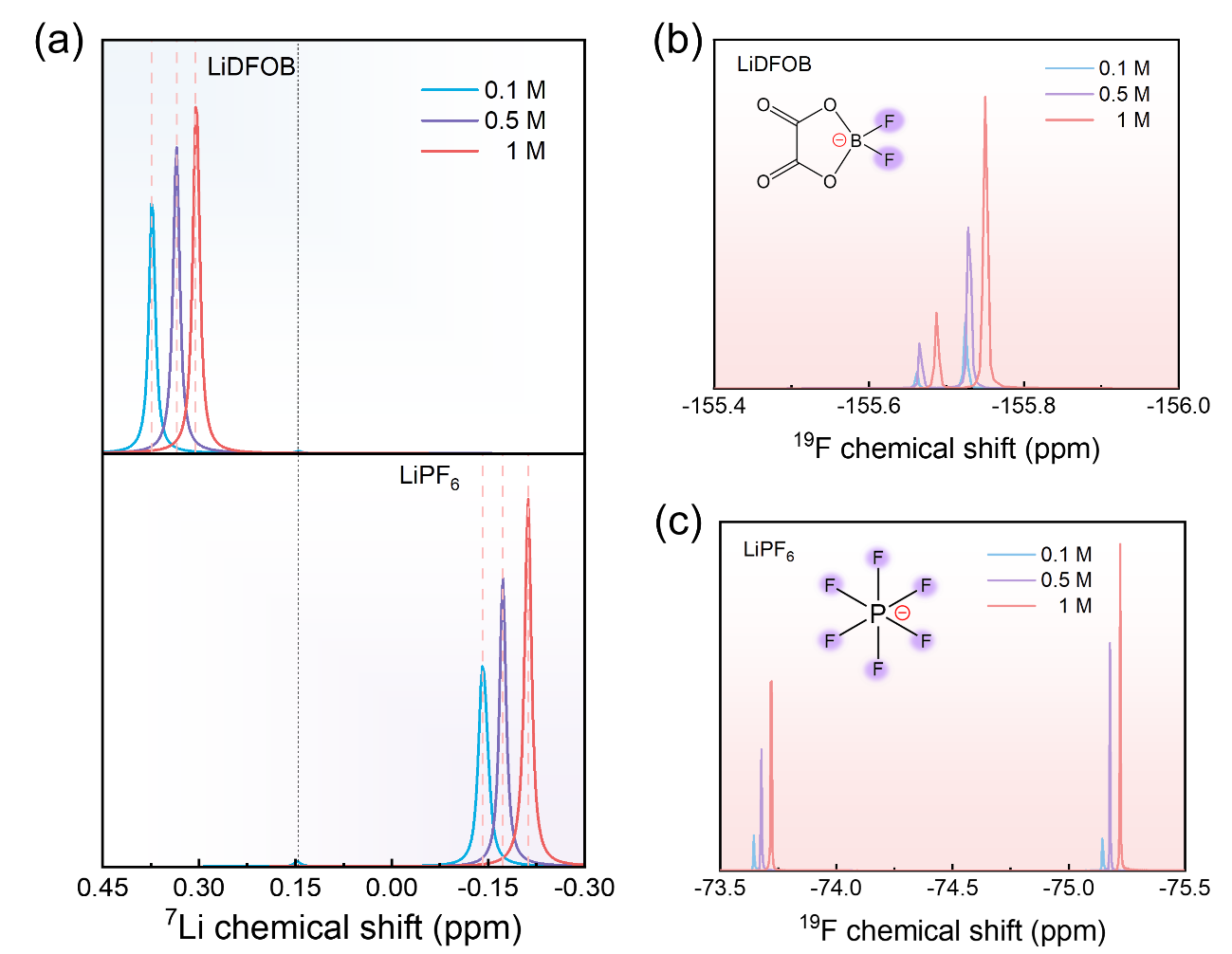


Figure S5. ^7^Li and ^19^F quantitative NMR spectroscopic analysis on electrolytes. (a) The introduction of DFOB^−^ causes a more positive upshift, indicating the significantly decreased electron cloud surrounding Li^+^ due to the electron-withdrawal effect that de-shields the ^7^Li nucleus. (b–c) Changes in the ^19^F spectra of different anions.


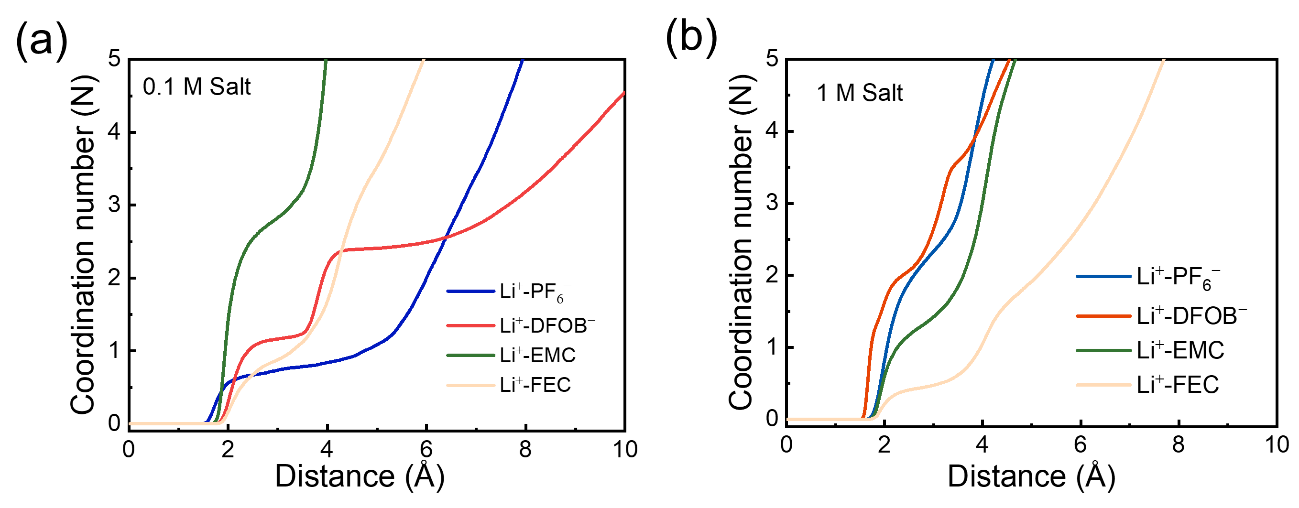


Figure S6. The coordination number of electrolyte species around Li^+^ in (a) 0.1 M and (b) 1.0 M.


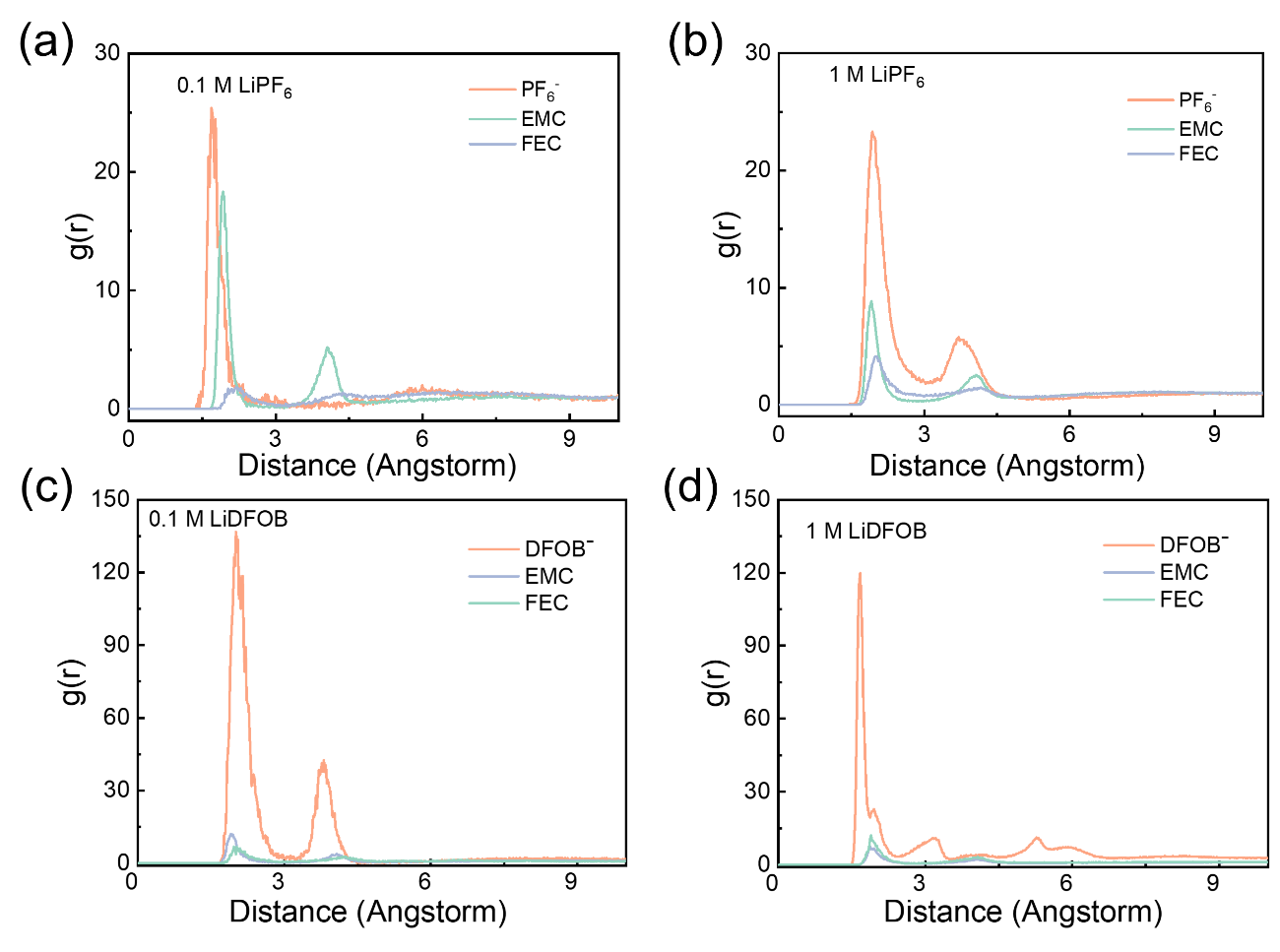


Figure S7. Radial distribution functions (RDF) in (a–b) LiPF_6_ based electrolyte and (c–d) LiDFOB based electrolyte.


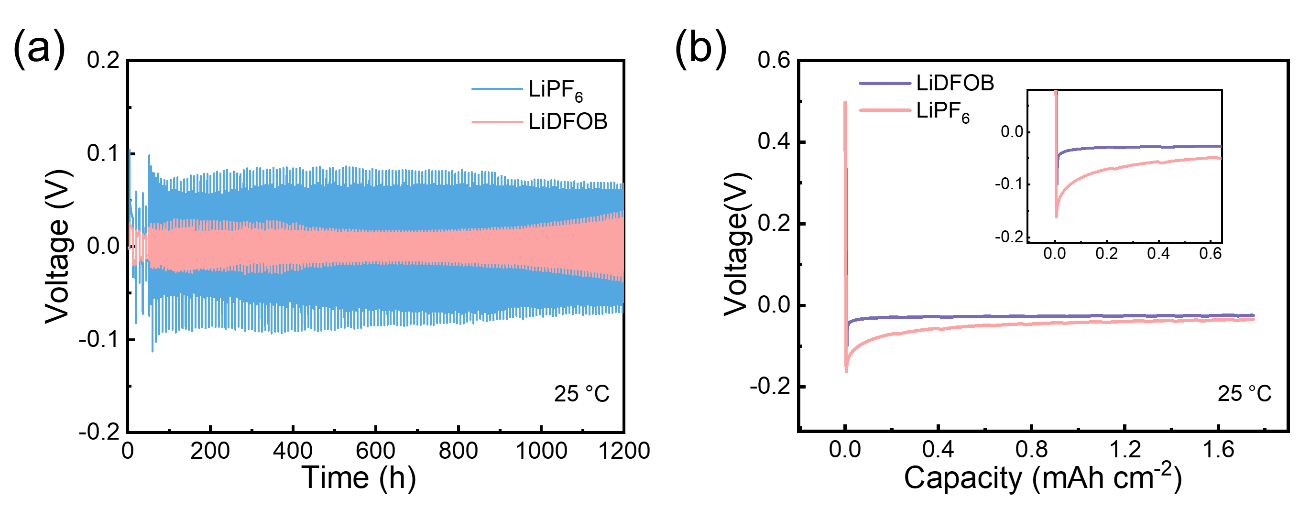


Figure S8. Measurement of overpotential with (a) Li||Li cell and (b) Li||Cu cell at 25 ºC.


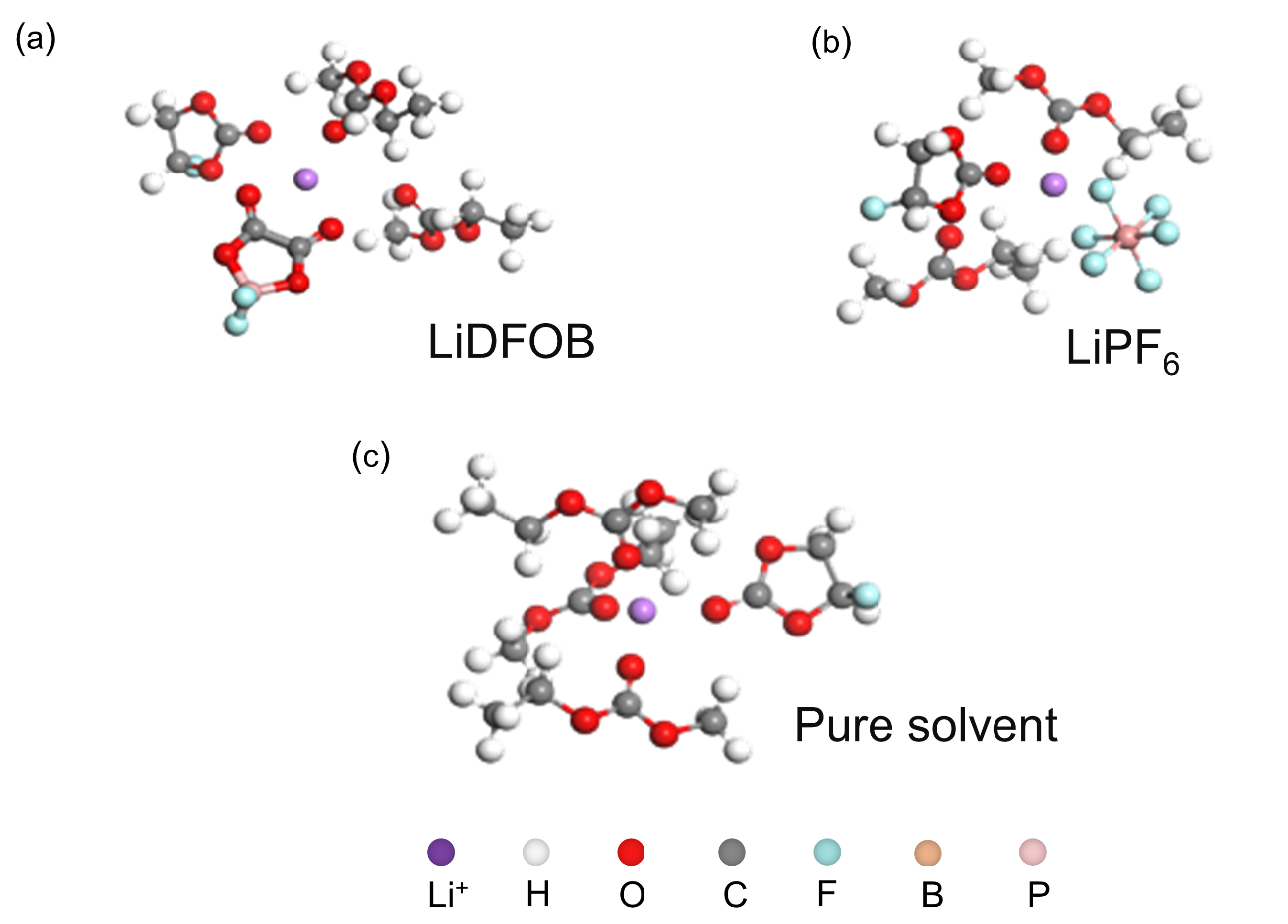


Figure S9. (a) (b) and (c) Li^+^ coordination structure with different solvation sheaths：DFOB^−^, PF_6_^−^ and neat solvents involved in the first solvation sheath.


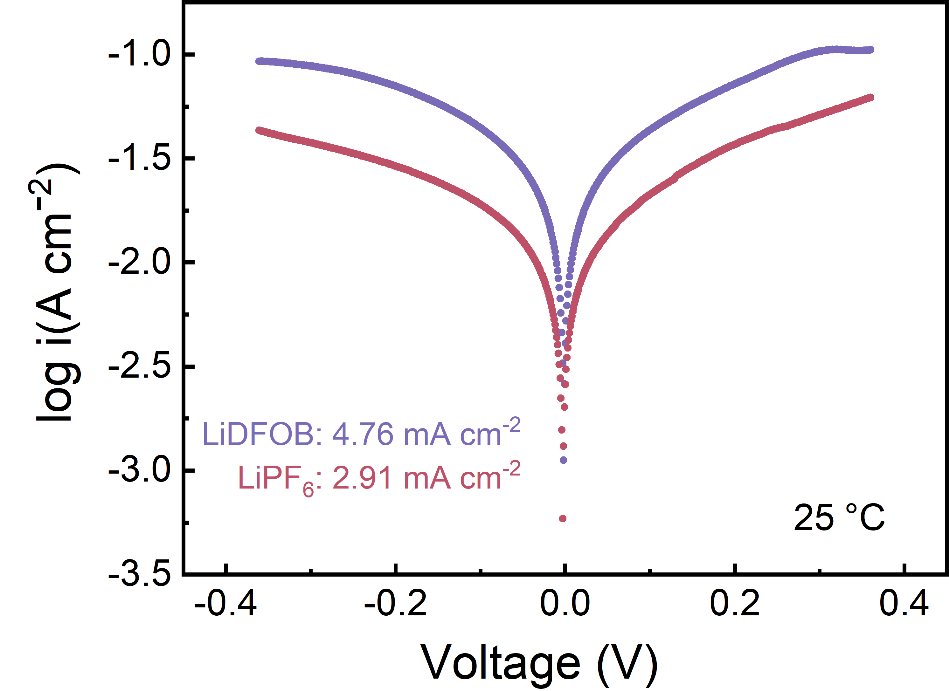


Figure S10. Tafel curves to derive exchange current density at 25 ºC.


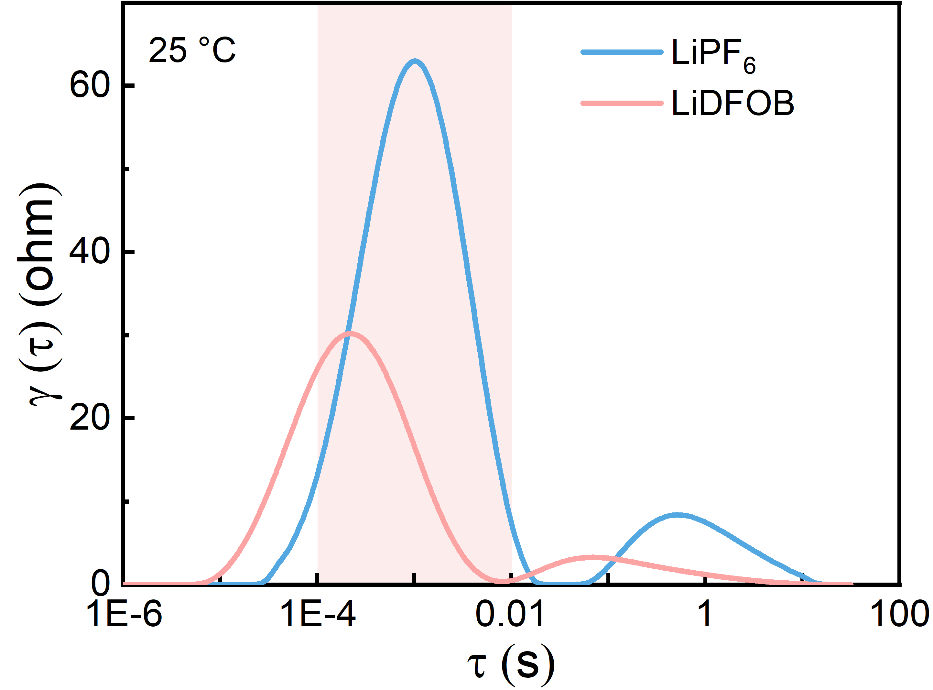


Figure S11. Decoupling kinetic features using the distribution of relaxation time (DRT) at 25 ºC.


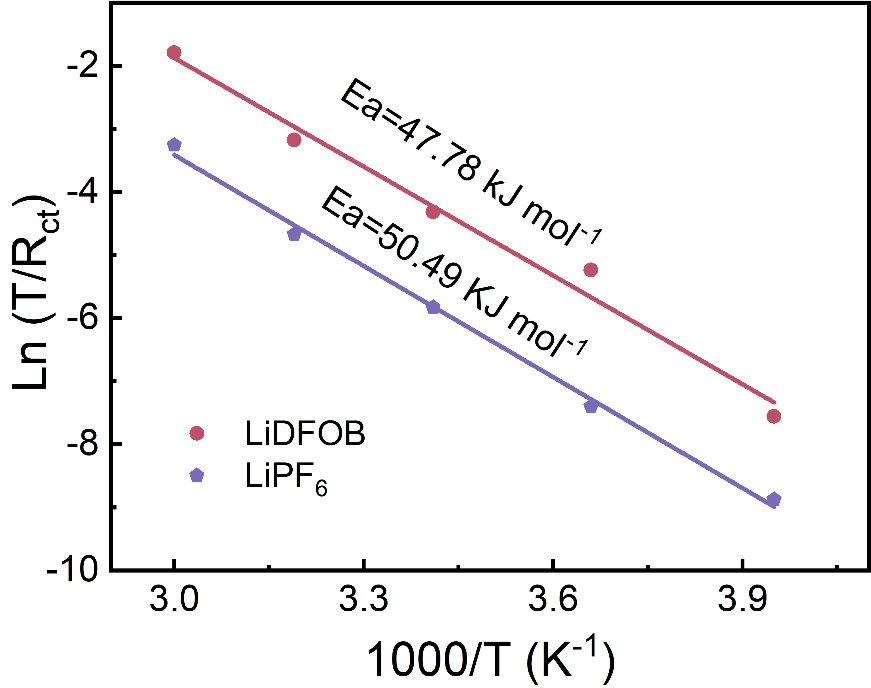


Figure S12. The activation energy of Li⁺ charge transfer obtained from the Arrhenius equation.


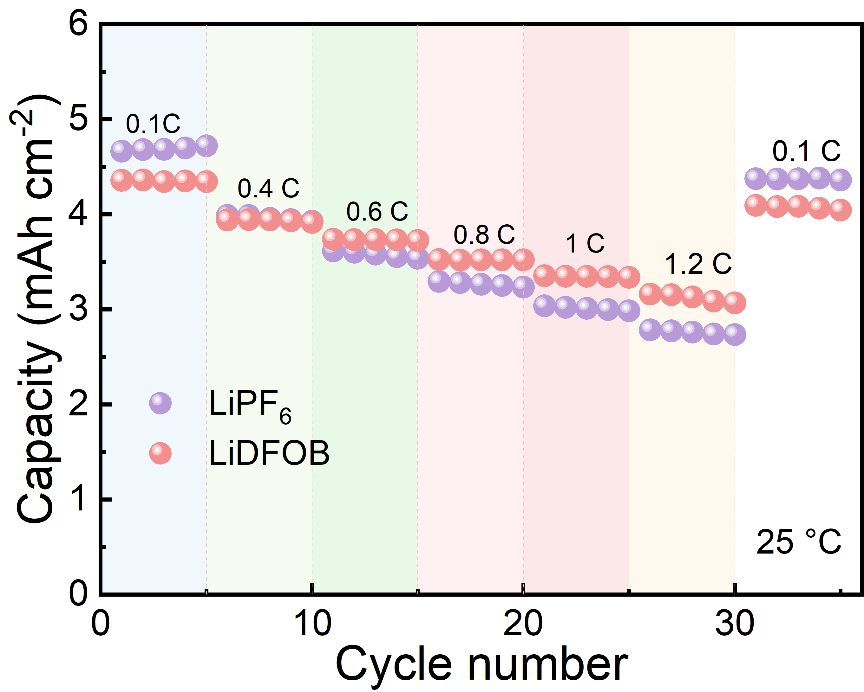


Figure S13. Rate capability of Li||NCM622 at 25 ºC.


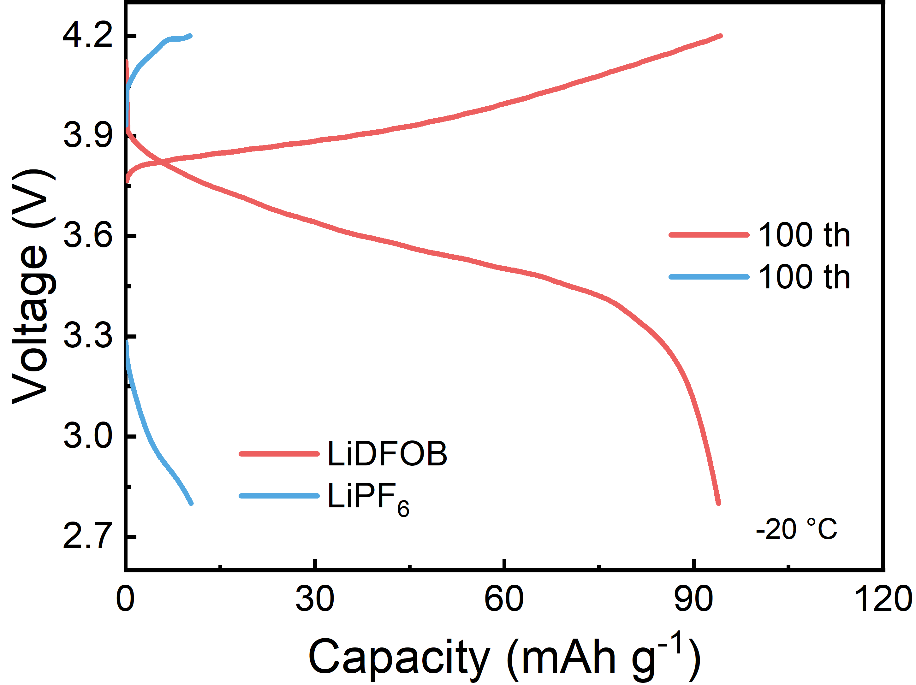


Figure S14. The typical charge/discharge curves of Li||NCM622 with LiPF_6_ based electrolyte at −20 ºC.


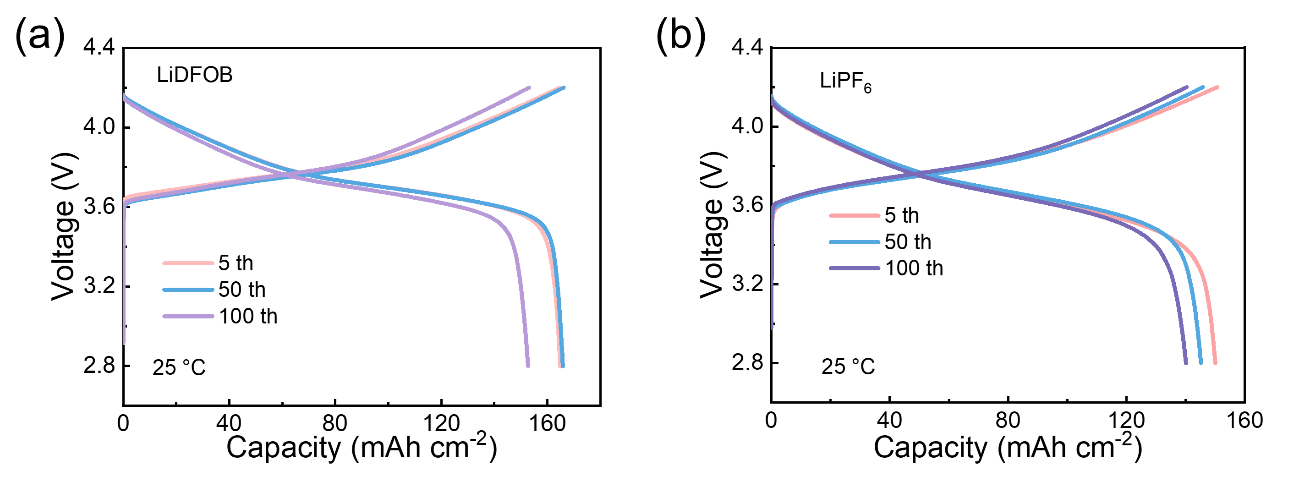


Figure S15. The typical charge/discharge curves of Li||NCM622 at 25 ºC with (a) LiDFOB and (b) LiPF_6_ based electrolytes.

Table S1 The step-by-step de-coordination energy of different electrolyte

| Electrolyte | Step 1 | Step 2 | Step 3 | Step 4 | |
| --- | --- | --- | --- | --- | --- |
| LiDFOB | 0.186 eV | 0.145 eV | 1.066 eV | | 6.069 eV |
| LiPF_6_ | 0.470 eV | 0.120 eV | 1.088 eV | | 6.031 eV |
| Pure solvent | 0.058 eV | 0.983 eV | 1.563 eV | | 2.081 eV |

Table S2 The value of R_ct_ from −20 ºC to 60 ºC

| R_ct_ | −20 ºC | | 0 ºC | 20 ºC | 40 ºC | 60 ºC |
| --- | --- | --- | --- | --- | --- | --- |
| LiDFOB | | 1916 Ω | 188 Ω | 75 Ω | 24 Ω | 6 Ω |
| LiPF_6_ | 7156 Ω | | 1640 Ω | 340 Ω | 107 Ω | 26 Ω |
